# Percutaneous Image-Guided Ablation of Renal Cancer: Traditional and Emerging Indications, Energy Sources, Techniques, and Future Developments

**DOI:** 10.3390/medicina61030438

**Published:** 2025-02-28

**Authors:** Vinson Wai-Shun Chan, Helen Hoi-Lam Ng, Khalil Abdulrauf, Hira Zaman, Aisha Ahmed, Jim Zhong, Tze Min Wah

**Affiliations:** 1 Leeds Institute of Medical Research, Faculty of Medicine and Health, University of Leeds, Leeds LS2 9JT, UK; 2 Division of Diagnostic and Interventional Radiology, Institute of Oncology, St James’s University Hospital, Leeds Teaching Hospitals NHS Trust, Leeds LS9 7TF, UK; 3 Pennine Care NHS Foundation Trust, Manchester OL6 7SR, UK; 4 School of Medicine, Faculty of Medicine and Health, University of Leeds, Leeds LS2 9JT, UK

**Keywords:** renal cell carcinoma, ablation techniques, interventional radiology

## Abstract

Percutaneous image-guided ablation (IGA) has emerged as an established alternative to surgical management for small renal masses. This comprehensive review examines traditional and emerging indications, energy sources, techniques, and future developments in IGA for renal cancer treatment. Traditionally, IGA has been indicated for frail or comorbid patients, those with solitary kidneys or chronic kidney disease, and those with histologically proven renal cell carcinomas less than 4 cm in size. Recent evidence supports expanding these indications to include T1b or T2 tumours and hereditary or recurrent renal cell carcinomas. The use of IGA combined with pre-ablation transarterial embolisation is discussed herein. This review then explores traditional energy sources including radiofrequency ablation, cryoablation, and microwave ablation, highlighting their respective advantages and limitations. Emerging technologies such as irreversible electroporation and histotripsy, as promising alternatives, are then presented, highlighting their advantage of being able to treat tumours near critical structures. Future research priorities highlight the need to establish high-quality evidence through innovative trial designs, as well as taking patient-reported outcome measures into account. Health economic considerations are key to ensuring that ablation therapies are cost-effective. The integration of artificial intelligence and radiomics shows vast potential for improving patient selection and treatment outcomes. Additionally, the immunomodulatory effects of ablative therapies suggest possible synergistic benefits when combined with immunotherapy which also require exploration in future research. Technological advancement and research developments will continue to broaden the role of IGA in clinical practice.

## 1. Introduction

Renal cell carcinoma (RCC) accounts for 3% of all cancer worldwide, with its incidence and mortality showing upward trends [1]. This rising incidence may be attributed to the increased use of cross-sectional abdominal imaging and subsequent detection of asymptomatic small renal masses (SRMs). SRMs are traditionally managed by radical nephrectomy (RN); however, robotic partial nephrectomy (PN) has developed into a mainstay of treatment for most SRMs. Regardless, PN is a complex surgery associated with high complication rates and a reduction in renal function [2,3].

Image-guided ablation (IGA) is an established alternative to PN or RN in managing SRMs. IGA utilises real-time imaging such as ultrasound, CT, or MRI to place needle probes surrounding the tumour, which is then destroyed using thermal energy such as radiofrequency ablation (RFA), microwave ablation (MWA), or cryoablation (CRYO). Non-thermal energy such as irreversible electroporation (IRE) can also be used. IGA is traditionally considered less invasive than PN, with lower complication rates and lesser reductions in renal function [4]. Non-inferiority compared to PN has also been demonstrated, albeit mainly in single-centre retrospective studies [4]. The American Urological Association (AUA) guidelines recommend clinicians to consider thermal ablation as an alternative approach in the management of cT1a solid renal masses less than 3 cm in size [5]. The European Association of Urology (EAU) guidelines recommend offering tumour ablation to frail and/or comorbid patients with SRMs, but with a low strength of evidence [1].

This article aims to be a comprehensive review of the traditional and emerging indications for IGA in renal cancer, as well as newly developing energy sources and techniques and areas for future developments.

## 2. Indications for Image-Guided Ablation

### 2.1. Traditional Indications

#### 2.1.1. Frail, Comorbid Patients

IGA has become a key treatment option for SRMs, particularly in patients who are not ideal surgical candidates due to advanced age, frailty, or significant comorbidities. Approximately 34% of all new cases of RCCs are diagnosed in individuals aged 75 and older, with incidence rates peaking in those between 85 and 89 years old [6]. Long-term data support the safety and efficacy of IGA in this group. A study conducted by Balageas et al. involved patients with a median age of 73.5 years who exhibited favourable oncological outcomes, a low complication profile, and the preservation of renal function following RFA [7]. Similarly, a retrospective analysis performed by Anglickis et al. comparing MWA and PN for SRMs in patients over the age of 70 years found no recurrences or metastases in either group, highlighting MWA as another viable treatment for elderly patients [8].

#### 2.1.2. Patients with Solitary Kidneys or Chronic Kidney Disease

Due to its superior nephron-sparing capabilities, IGA is also indicated for patients with a solitary kidney or chronic kidney disease (CKD) [9]. An analysis from the European EuRECA registry, which included 70 patients with a solitary kidney, demonstrated that image-guided CRYO is a safe treatment option for this group [10]. The mean reduction in the estimated glomerular filtration rate (eGFR) was only 6.2 mL/min/1.73 m^2^, with no patients experiencing worsening CKD or requiring dialysis. A retrospective analysis in Japan of patients with stage 4 or 5 CKD demonstrated good oncological outcomes and an acceptable mean decrease in renal function of 5.0 ± 4.0 mL/min/1.73 m^2^ at 24 months [11]. Notably, one patient did require maintenance dialysis at 21 months [11]. Compared to PN, CRYO showed comparable renal function preservation, complication rates, and recurrence rates in patients with solitary kidneys, reaffirming the role of IGA in managing small RCCs in this population [12].

#### 2.1.3. Histologically Proven Renal Cell Carcinoma

In patient selection, it is crucial to ensure that a biopsy is performed prior to treatment as a separate session and that only patients with malignant histology are treated. The benign histology rate is currently as high as 30% in patients undergoing PN [13,14] and 20.9% in CRYO [15]. Despite its potential benefits, pre-treatment biopsy remains a non-standard practice in some centres. An analysis of the European EuRECA registry revealed that pre-CRYO biopsy was associated with a significantly lower rate of benign histology compared to cases without pre-CRYO biopsy [15]; this has been reflected in a change in the EAU guidelines, which now recommend performing a percutaneous renal mass biopsy prior to, and not concomitantly with, thermal ablation [1]. While there are concerns surrounding the seeding and non-diagnostic rates of percutaneous renal tumour biopsy, the opposite is demonstrated in experienced centres and systematic reviews, showing high diagnostic rates and minimal seeding rates [16,17]. Furthermore, pre-treatment biopsies also contribute to a more homogenous patient cohort, which helps standardise research populations and improve the reliability of outcomes [18].

#### 2.1.4. Size and Location of Tumours

Traditionally, exophytic and posterior tumours less than 4 cm in diameter are deemed to be the least challenging when performing IGA, due to the distance from other vital structures such as the bowel, ureter, or kidney collecting system. A retrospective analysis by Luzzago et al. found that RFA- and MWA-treated tumours larger than 3cm are at increased risk of not achieving trifecta (defined by an absence of major complications, complete ablation at 6 months, and a less than 30% decrease in eGFR) and have higher local recurrence rates when compared to those smaller than 3 cm [19]. Due to the ability to visualise the ice ball and treatment margins, CRYO, on the other hand, can generally be used for tumours up to 4 cm in size, or for even larger tumours in selective cases where other treatment options are not feasible [20,21].

Endophytic or hilar tumours are usually more challenging to treat due to their proximity to vital structures such as renal vessels, the collecting system, and the bowel. The heat-sink effect, caused by thermal energy being dissipated by blood flow in nearby vessels, further reduces the technical success rate of thermal ablation [22]. This is occasionally mitigated by a longer treatment time [23]. In tumours that are close to vital structures, counter-strategies such as pre-ablation ureteric stent insertion [24], cold pyeloperfusion [25], and hydrodissection [26] have been utilised. However, the percentage of patients with endophytic tumours achieving trifecta remains low at 58.8% vs. 65.3% in PN [27]. IGA should only be performed in these challenging cases where other operative measures are not suitable.

The sizes and locations of tumours are integrated into scoring systems such as the R.E.N.A.L. (Radius, Endophytic/Endophytic, Nearness to collecting system or renal sinus, Anterior/posterior location, location relative to polar lines) nephrometry score [28] and the PADUA (Preoperative aspects and dimensions used for an anatomical) classification [29] to predict treatment success and outcomes in PN. Their roles, however, may be limited in IGA [30,31]. Similarly, scoring systems developed specifically for IGA, such as the P-RAC score [32] and the (MC)2 score [33], have also been found to have limited use in predicting treatment success on external validation [34,35].

### 2.2. Emerging Indications

#### 2.2.1. T1b or T2 Tumours

In patients for whom other alternative treatment modalities are not appropriate, IGA, especially CRYO, is often employed in an attempt for disease control, with reports of CRYO being performed in T1b or even T2 tumours. A retrospective review by Atwell et al. of 46 treated RCCs in the range of 4.1–6.4 cm found an estimated progression-free survival rate of 96.4% at 3 years after treatment [36]. Long-term data also suggest a cancer-specific survival rate of up to 96.4% at 10 years in patients undergoing image-guided cryoablation for T1b tumours [21]. Image-guided ablation for T2 tumours, less commonly performed, was found by Moyanagh et al. to achieve a 100% recurrence-free survival rate at 3 years [37].

One suggested strategy to manage large RCCs is pre-ablation transarterial embolisation (TAE). The EAU guidelines [1] only acknowledge the use of TAE in candidates unsuitable for surgery and as a palliative measure to manage RCC-associated haematuria or pain. The mechanism of TAE is said to be two-fold: firstly by tumour size reduction due to vessel occlusion, thus reducing tumour perfusion, and secondly by a reduction in hypervascularity to mitigate haemorrhage, especially in larger or central RCCs [38,39]. TAE also reduces the heat sink effect caused by surrounding vessels, thus improving the efficacy of thermal ablation [39]. Several studies have also suggested the use of ethiodised oil during TAE to improve visualisation of the RCC during CT-guided ablation [40,41], allowing for more secure ablation margins and needle placement accuracy. This has been shown to improve local tumour control, particularly for larger RCCs and those near critical structures [40,41].

When compared to ablation alone, TAE with ablation was found to have a 100% secondary technical success rate, despite being associated with higher R.E.N.A.L. nephrometry scores; the ablation-alone group achieved an 84.8% secondary technical success rate [42]. Therefore, TAE can also be used to reduce the need for multiple sessions of ablation, particularly for larger T1b/T2a tumours, which have a higher likelihood of local recurrence [42]. While the technique is performed in practice, technical standards such as the timing between embolisation and ablation have yet to be established; the results of the EMBARC trial are highly anticipated to provide further information regarding the technical feasibility and safety of pre-ablation embolisation [43].

#### 2.2.2. Recurrent or Hereditary RCCs

Hereditary RCCs account for 2–4% of all RCC cases [44] and are associated with von Hippel–Lindau syndrome (VHL), hereditary leiomyomatosis and renal cell cancer (HLRCC), hereditary papillary renal carcinoma (HPRC), and Birt–Hogg–Dube syndrome (BHD) [45]. Due to the recurrent and bilateral nature of these RCCs, optimising oncological control and renal function preservation proves to be a challenge. Repeated PN in multifocal and recurrent RCCs is also technically challenging, associated with high complication rates and a need for haemodialysis [46,47]. IGA has proven to be a viable option for these patients. An analysis of the EuRECA registry reported a technical success rate of 99% and an overall complication rate of 1.7% with excellent renal function preservation [45]. The 5-year cancer-specific survival rate was 90.9% [45]. It is remarkable to note that a long-term follow-up spanning over 17 years of 17 VHL patients who underwent RFA or CRYO for renal tumours demonstrated a 100% cancer-specific survival rate at 10 years, with none requiring dialysis [48].

## 3. Energy Sources

### 3.1. Traditional Energy Sources

#### 3.1.1. Radiofrequency Ablation (RFA)

RFA for renal cancer was first performed in 1997 [49]. The high-radiofrequency energy causes ionic agitation, generating frictional heating leading to cancer cell necrosis at a temperature of above 60 °C [50]. RFA was the first modality to be used widely in IGA and is still widely used as the standard of care in some cases, although it is being slowly replaced by new energies such as CYRO and microwave energy. It is widely known that RFA suffers from some limitations, such as an inability to visualise the treatment margins intraoperatively, as well as being significantly prone to heat-sink effects [23]. This can lead to increased treatment times and less predictable ablation zones, which could be factors of reduced technical success [51]. Long-term follow-ups of RFA have suggested optimal oncological control of local recurrence survival and cancer-specific survival rates of over 95% at 5 years [21].

#### 3.1.2. Cryoablation (CRYO)

CRYO utilises argon gas to create the Joule–Thompson effect: the high-pressure argon gas undergoes rapid expansion through a valve, leading to rapid cooling and forming an ice ball at the end of the probe [52]. This allows for a significant advantage compared to other modalities, which is the ability to visualise the ice ball and, thus, the treatment margins in real time intraprocedurally [53]. This allows for a visual confirmation of complete treatment response and the avoidance of critical structures to reduce the risk of major complications. In addition, multiple needle probes can be used with CRYO to achieve a predictable and symmetrical area of the ablation zone, allowing for it to be utilised in larger tumours, with reports of CRYO being used in T1b tumours [42] or even T2 tumours in selected cases [37]. In a Japanese multi-centre study of 46 patients with T1b tumours, CRYO was found to have a significantly higher primary technical success rate compared to RFA (96% vs. 65%) [54]. In T1a tumours, CRYO achieved excellent long-term oncological outcomes with a 100% cancer-specific survival rate observed at 10 years [21]. This figure was slightly lower in T1b tumours, at 96.4% at 10 years [21].

#### 3.1.3. Microwave Ablation (MWA)

MWA utilises electromagnetic waves to heat up water molecules within cells to directly cause cellular destruction [55]. When compared to RFA, although the mechanism of cellular death is similar, MWA achieves higher intra-tumoral temperatures and larger ablation zones within a shorter ablation time [56]. This is likely due to MWA being less limited by heat-sink effects compared to RFA. MWA also produces less charring and lesser tissue boiling effects when compared to RFA, which allows for better thermal conductivity through tissues, allowing for more predictable ablation zones [56]. However, the high thermal energy from MWA in theory increases the risk of thermal injury to adjacent critical structures [56]. In a multi-centre study of over 800 patients, MWA was found to have significantly shorter treatment times compared to RFA and CRYO but no significant difference in terms of complication rates or oncological outcomes in SRMs [57].

#### 3.1.4. Comparison of the Traditional Modalities

While RFA, CRYO, and MWA are all commonly used modalities for ablating SRMs, the evidence comparing the modalities is limited. A large multi-centre retrospective study in Italy compared MWA to RFA in the treatment of SRMs and found significantly higher achievement rates of trifecta and shorter operative times in patients who underwent MWA compared to RFA [58]. However, another large retrospective study in the United States comparing 297 patients undergoing CRYO, MWA, and RFA found no significant difference in technical success rates, oncological outcomes, complication rates, or changes in renal function amongst the three treatment modalities [59].

### 3.2. Emerging Energy Sources

#### 3.2.1. Irreversible Electroporation (IRE)

**Figure 1:**
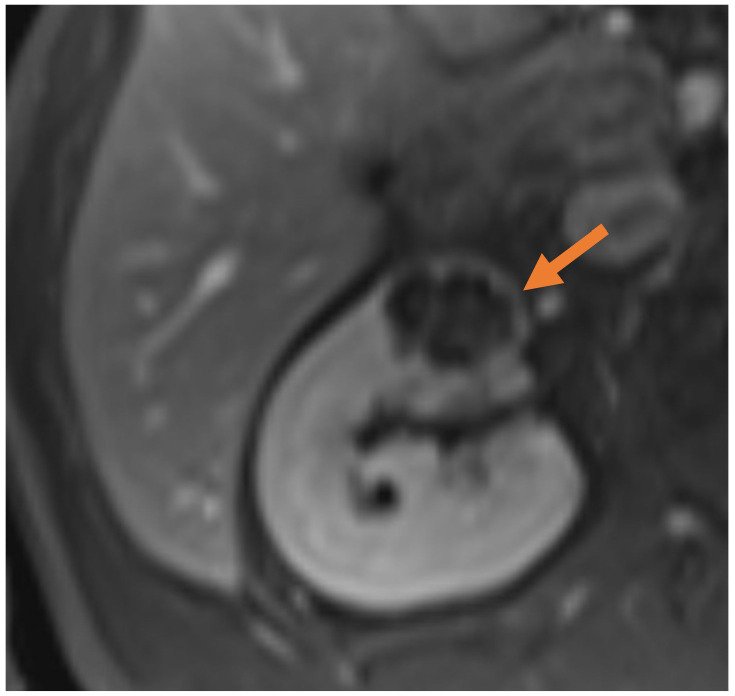
Axial view of contrast-enhanced T1-weighted MRI showing a partly cystic and peripherally enhancing lesion in the anterior right kidney (arrow).

**Figure 2:**
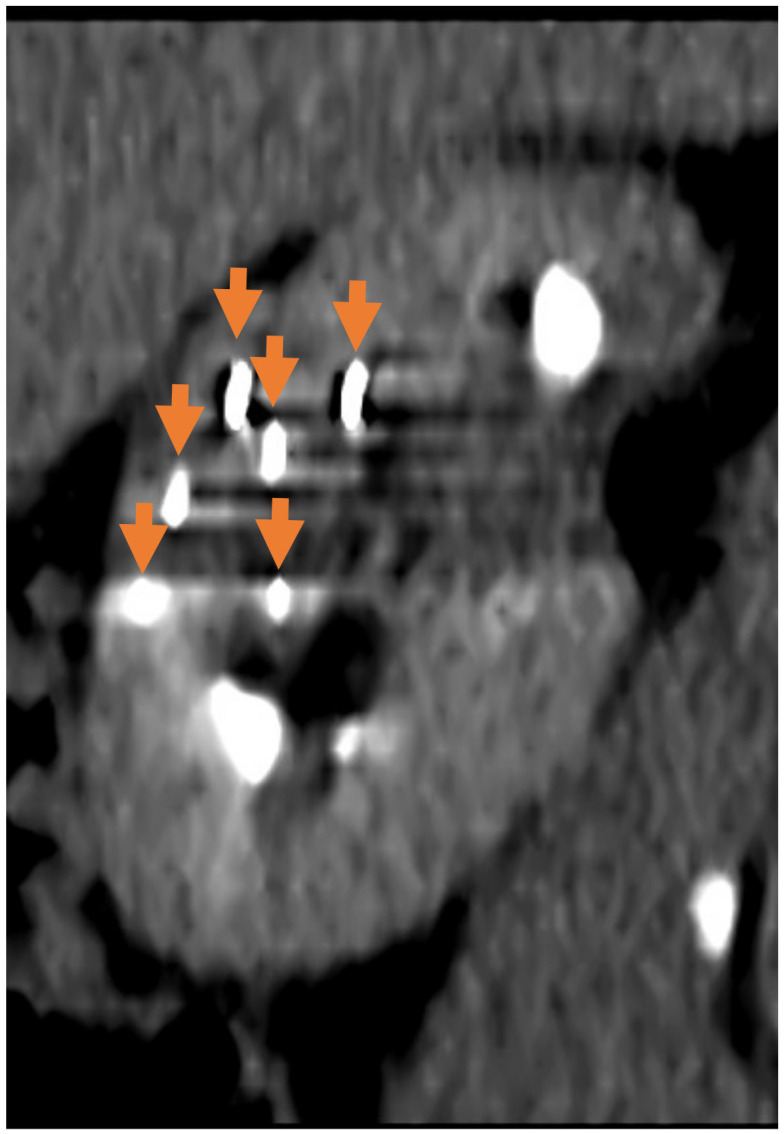
Intraoperative CT (sagittal view) showing 6 IRE needles (arrows) bracketing the tumour.

IRE or Nanoknife (Nanoknife^®^, AngioDynamics, Latham, NY, USA) is a non-thermal ablative technology where high-voltage electrical impulses lead to irreversible nanopores in cellular membranes, disrupting cellular functions and leading to cell death [60]. The unique feature of IRE is the ability to spare collagen-rich structures, making it an ideal modality to treat tumours that are closely abutted to critical structures such as the collecting system, ureter, or blood vessels. The non-thermal nature of IRE also minimises the heat-sink effect, increasing the efficacy of treatment in comparison to other modalities. Figure 1 and Figure 2 show an example of IRE treatment in a patient with an anterior mass of the right kidney that was close to the hilar structures.

IRE is currently reserved as a problem-solving technology, with the first case performed in 2011 by Pech et al. [61]. Since then, a mid-term experience of 30 patients achieved a primary technical success rate of 73.3% and an overall technical success rate of 97% [62]. The same study showed good oncological outcomes, with a local recurrence-free survival rate of 91% at a 3-year follow-up and only one major complication. Another study showed primary technical success rates of up to 91.7% and a 5-year metastasis-free survival rate of 97.1% [63]. The definitive role of IRE in the treatment of SRMs is yet to be determined, but it is currently strong as an effective problem-solving technique in tumours where other ablative methods are less ideal or inappropriate for the patient.

#### 3.2.2. Histotripsy

Histotripsy is a novel therapeutic technique that utilises focused ultrasound waves to mechanically destroy tissue without ionising radiation [64]. It is non-thermal and thus does not have the risk of thermal damage seen in traditional IGA modalities [65,66]. This reduces collateral damage, complication rates, and recovery times [67]. Furthermore, histotripsy has recently received FDA approval for the treatment of liver malignancies, based on the #HOPE4LIVER study [68]. The world’s first histotripsy in kidney tumours was performed as part of the CAIN (the HistoSonics System for treatment of primary solid renal tumors using histotripsy) trial (NCT05432232) [69,70], with the #HOPE4KIDNEY (The HistoSonics Edison System for Treatment of Primary Solid Renal Tumors Using Histotripsy) trial (NCT05820087) also ongoing to evaluate the use of histotripsy in renal cancer.

## 4. Future Research Priorities and Directions

### 4.1. Establishing the Role of IGA in SRMs

While IGA is a well-established management option for SRMs, the current evidence base is predominantly derived from retrospective cohort studies, with limited prospective or randomised data. The challenges in conducting high-quality trials in this field are evident from the recent SURAB feasibility study [71] and the CONSERVE trial [72] both failing to recruit and randomise the targeted number of patients. These recruitment difficulties highlight the need for innovative trial designs and strategies to overcome patient accrual barriers. A key future research priority should be the development and execution of a level 1 evidence trial to definitively establish the role of IGA in managing SRMs.

The NEST (Nephron Sparing treatment) study is a highly anticipated trial employing a novel cohort-embedded randomised control trial (RCT) design in an attempt to overcome the traditional recruitment challenges of an RCT [73]. In the NEST feasibility study, a total of 200 patients were recruited into the cohort, and 50 were successfully enrolled into the RCT, with a consent rate of 84% in the image-guided CRYO arm. Whilst not powered to show differences between PN and CRYO, the initial findings found CRYO to be associated with shorter hospital stays, lower complication rates, and similar renal function preservation. The full NEST trial is highly anticipated, with the potential to provide level 1 evidence in this field.

On the other hand, it is also important to consider indirect outcomes such as patient-reported outcome measures (PROMs). The assimilation of PROMs as an endpoint in clinical studies is a crucial area for the future development of IGA in renal cancer. Despite their common use, a recent systematic review revealed significant gaps in the existing reports of PROMs in renal ablation [74]. The review identified that only eight studies investigated PROMs in the context of renal ablation, with the 36-item Short-Form Survey (SF-36) being the most frequently employed outcome measure tool. The studies assessed patients’ social, functional, and physical well-being over follow-up periods ranging from one day to six months. The authors found that that patients’ quality of life was positively influenced by ablation versus non-ablative treatments [74]. There is a need for more prospective studies collecting general and disease-specific PROMs to assess renal tumour ablation outcomes. Increasing research to address this gap will help to provide patient-centred care and vital insights into factors affecting patient recovery and satisfaction post-ablation.

An important factor to consider when evaluating the role of IGA in managing renal cancer is its health economics and overall cost to the healthcare system. The existing literature often associates IGA with lower costs compared to PN. For example, a Medicare study [75] reported significantly lower direct treatment costs for IGA (USD 7988) compared to PN (USD 18,359). A decision-analysis model constructed by Wu et al. also reported image-guided CRYO to be more cost-effective than PN in specific outcome scenarios [76]. However, larger-scale studies with extended follow-up are needed to fully assess the economic impact, particularly in terms of quality-adjusted life years (QALYs). These considerations should be incorporated into future trials and prospective study designs.

### 4.2. Intraprocedural Imaging

IGA of small renal masses is often performed under CT- or ultrasound-guided assistance, but ablation can also be performed with MRI guidance, which has a few benefits including being radiation-free. Firstly, compared to CT, MRI offers superior contrast resolution, coupled with the diverse array of sequences available, enabling superior characterisation and localisation of tumours, particularly in cases of endophytic and hilar tumours or poorly enhancing tumours that can be challenging to visualise on CT or ultrasound [77,78]. Furthermore, MRI allows for potential real-time control of needle advancement [78]. However, these theoretical advantages have yet to translate into clinical benefits, as shown in a retrospective propensity-matched analysis of patients undergoing MRI- or CT-guided CRYO. Other than a significantly longer procedural time in the MRI group, no significant difference were noted in terms of complication rates, eGFR decline, or oncological outcomes [78]. Furthermore, MRI-guided ablation is likely to be significantly more expensive compared to CT-guided ablation. This is because of the prolonged procedural time and the need for an MRI-compatible instrument. Additionally, a significant portion of patients with implanted metalwork such as pacemakers may not be suitable for MRI-guided ablation, or the imaging may be obscured by artefacts. Given the limited significant benefit of MRI-guided ablation compared to CT-guided ablation, it is unlikely that performing MRI-guided ablation as a standard of care would be cost-effective. However, further prospective studies are necessary to determine the most suitable intraprocedural imaging modality for ablation targeting.

Another exciting development in the field of intraprocedural imaging is the increasing availability of fusion imaging, which combines two or more modalities. For instance, a preprocedural image like contrast-enhanced CT or MRI is fused with an intraprocedural modality like ultrasound or cone beam CT. Fusion imaging combines the advantages of multiple modalities, such as ultrasound’s real-time guidance of needle movement and CT/MRI’s three-dimensional visualisation of the needle and the target lesion, possibly reducing the risk of complications and providing a better evaluation of treatment margins [79,80,81]. The development of positron emission tomography (PET) scans also allows for PET/CT fusion imaging during the ablation procedure, providing tumour visibility throughout procedures even when intraprocedural CT tumour visibility is low [82]. Furthermore, with the increasing availabilities of artificial intelligence (AI) and software registration, fusion imaging will possibly become the standard of care in IGA in the near future.

### 4.3. Radiomics and Artificial Intelligence (AI) in IGA

The application of AI in IGA remains an underexplored area, with its potential largely centred on two key aspects. Firstly, while existing scoring systems like the R.E.N.A.L. nephrometry score are widely used, their ability to predict treatment outcomes is limited. Radiomics and machine learning, already employed in other cancers and treatments, such as IGA for hepatocellular carcinoma, have demonstrated potential in predicting treatment response and complications [83]. These tools could help identify treatment responders and non-responders, as well as patients at higher risk of complications, enabling clinicians to consider surgery or active surveillance [84]. Secondly, the use of AI and robotic systems can standardise treatments and improve outcomes of IGA. The latest Edison histotripsy manufactured by Histosonics^®^ features a single robotic arm to manoeuvre the treatment probe in multiple directions to ensure consistency and flexibility when planning every treatment [68,85]. Furthermore, navigational technologies are beginning to utilise AI and image fusion to improve the accuracy and technical success of procedures [86]. The future of radiomics and AI for IGA is vast and exciting; however, further research is required to determine whether AI in IGA can improve clinical outcomes.

### 4.4. Immunomodulation and Potential Combination with Immunotherapy

Ablative therapies have been hypothesised to induce the abscopal effect, which emerges from a systemic immune response that results in the regression of an untreated distant or local tumour following the treatment of a primary or metastatic tumour. This phenomenon is attributed to multiple immunomodulatory processes. Firstly, ablative therapy can directly eradicate pro-tumour cells, such as M2-like phenotype macrophages, or it can convert naïve macrophages or M2-like phenotypes to more pro-inflammatory M1 phenotypes. This transformation directly contributes to creating a more hostile tumour microenvironment, hindering the progression of cancer cells [87,88]. Secondly, ablation was also found to stimulate the release of pro-inflammatory cytokines and chemokines such as Interferon gamma (IFNγ); these cytokines and chemokines further shift the tumour microenvironment from “cold” (immunosuppressive) to “hot” (immunostimulatory) and facilitate the recruitment of antigen-presenting cells (APCs) [87,89]. The ablative treatment leads to the release of damage-associated molecular patterns (DAMPs) such as heat shock proteins, calreticulin, and high mobility group box 1. These DAMPs activate the innate immune system alongside the released chemokines and cytokines and facilitate the recruitment of APCs such as dendritic cells, neutrophils, natural killer cells, and macrophages [90]. These APCs take up antigens released through the process of ablation, including microfragments of the ablated tumour, and migrate to lymph nodes and the spleen in order to present these antigens to CD8+ cytotoxic T-cells and CD4+ helper T-cells. These antigen-specific primed T-cells then travel in circulation to reach untreated tumours, allowing them to directly engage in the lysis of distant or local malignant cells, triggering the abscopal effect [87,91,92,93,94,95].

This hypothesis was tested by our group by utilising plasma from CRYO-treated patients with RCC; we found a significant increase in immune markers such as serum cytokine and inflammatory proteins in patients undergoing CRYO and RFA [96]. Preliminary clinical data further support this potential. For instance, a pilot study by Campbell et al. evaluated tremelimumab with or without CRYO in 30 patients with metastatic RCC and showed promising safety data, yet no significant differences in progression-free survival were observed [97]. In other cancers, such as breast or liver, studies of synergistic therapies remain largely confined to phase I and II trials. In one study of unresectable primary liver cancer, Yang et al. found the progression-free survival to be significantly longer in patients who receive IRE combined with allogenic natural killer cells in comparison to IRE alone (15.1 months vs. 10.6 months) [98].

Exploring the immunomodulation ability of histotripsy is one of the secondary objectives of the HOPE4LIVER trial [68] and the CAIN trial [69,70]. While CRYO and RFA have demonstrated the ability to stimulate immune responses, this effect is believed to be even more pronounced with histotripsy, owing to its mechanical destruction of tumour cells and preservation of antigens [87]. This is, in particular, supported by the world-first THERESA pilot study [99] investigating histotripsy for liver tumours, where the treatment of colorectal liver metastases using the histotripsy system induced size reductions in non-treated lesions across different liver lobes, with a sustained reduction in the CEA tumour marker [100].

While the immunomodulation and abscopal effects show promise, there are no reports of complete regression for either local or remote metastasis after ablative therapies. Therefore, combining these therapies with other approaches like immune checkpoint inhibitors is necessary to achieve a robust systemic response that can induce full cancer regression. However, this approach has only been tested in murine models using CTLA-4 and PD-L1 in combination with mechanical high-intensity focal ultrasound, which resulted in complete regression of the contralateral tumour after treatment of the primary tumour [101].

Although preliminary data are encouraging, further research is needed to elucidate the synergistic potential of IGA and immunotherapy, whether through basic science studies or through future translational clinical trials.

## 5. Conclusions

Traditionally, IGA for renal cancer has been limited to small, exophytic tumours in frail or non-surgical candidates. However, its indications have expanded to include larger and more complex tumours, driven by advancements in technologies such as IRE and by combining ablation with TAE. Emerging techniques like histotripsy hold significant promise, alongside the anticipated development of level 1 evidence supporting IGA for small renal masses. Additionally, the integration of AI and the exploration of combination therapies, particularly in the management of advanced or metastatic renal cancer, are expected to further broaden the role of IGA in clinical practice.

## Abbreviations

The following abbreviations are used in this manuscript:

RCC	Renal cell carcinoma
SRM	Small renal mass
RN	Radical nephrectomy
PN	Partial nephrectomy
IGA	Image-guided ablation
RFA	Radiofrequency ablation
MWA	Microwave ablation
CRYO	Cryoablation
IRE	Irreversible electroporation
AUA	American Urological Association
EAU	European Association of Urology
CKD	Chronic kidney disease
eGFR	Estimated glomerular filtration rate
R.E.N.A.L.	Radius, Endophytic/Endophytic, Nearness to collecting system or renal sinus, Anterior/posterior location, location relative to polar lines
PADUA	Preoperative aspects and dimensions used for an anatomical classification
TAE	Transarterial embolisation
VHL	von Hippel–Lindau syndrome
HLRCC	Hereditary leiomyomatosis and renal cell cancer
HPRC	Hereditary papillary renal carcinoma
BHD	Birt–Hogg–Dube syndrome
CT	Computed tomography
MRI	Magnetic resonance imaging
AI	Artificial intelligence
QALY	Quality-adjusted life year
PROM	Patient-reported outcome measure
SF-36	36-item Short-Form Survey
